# Assessment of pressure pain thresholds in collisions with collaborative robots

**DOI:** 10.1371/journal.pone.0215890

**Published:** 2019-05-02

**Authors:** Moon Young Park, Doyeon Han, Jung Ho Lim, Min Kyung Shin, Young Rok Han, Dong Hwan Kim, Sungsoo Rhim, Kyung Sook Kim

**Affiliations:** 1 Department of Biomedical Engineering, College of Medicine, Kyung Hee University, Seoul, Korea; 2 Department of Mechanical Engineering, Kyung Hee University, Gyeonggi-do, Korea; 3 Department of Dermatology, College of Medicine, Kyung Hee University, Seoul, Korea; 4 Department of Physical Medicine and Rehabilitation, College of Medicine, Kyung Hee University, Seoul, Korea; Huazhong University of Science and Technology, CHINA

## Abstract

In recent years, safety issues surrounding robots have increased in importance, as more robots are in close contact with humans, both in industrial fields and elsewhere. Safety standards for industrial robots operating in specific spaces have been established, but no such standards have been specified for collaborative and service robots. To establish safety standards for such robots, we assessed pressure pain thresholds for collisions between humans and robots, under the assumption that the pain threshold is lower than the mild injury threshold. The pressure pain threshold for collision with a robot was measured in 90 male Korean adults using a homemade collision system. The pain thresholds were measured three times at 15 sites, including the forehead. The highest threshold was 196.1 ± 85.8 N/cm^2^ at the back of the hand, and the lowest was 65.1 ± 22.6 N/cm^2^ at an arm nerve. Moderate thresholds, i.e., 100–120 N/cm^2^, were noted on the forehead, neck muscle, ball of the thumb, and shin. The thresholds of participants < 30 years of age were lower, by 3–33%, than those of participants aged > 30 years. Thresholds differed by body mass index only at certain sites, including the shoulder joint, neck, and back of the hand. The pressure pain threshold depended on individual characteristics, body site, and age. The threshold relevant to potential human-robot collisions was determined to be between 65.1 ± 22.6 and 196.1 ± 85.8 N/cm^2^.

## Introduction

Robots are complex machines that perform behaviors or tasks with a high degree of autonomy. They have been used mainly in industry to automate production processes. Industrial robots have tended to be stationary, operating in specific spaces and performing repetitive and limited actions. In recent years, collaborative robots have been introduced into the industrial sector. A collaborative robot operates in close cooperation with humans [1]. Such robots share the workplace, and perform tasks simultaneously, with humans during production operations. In addition, service robots have been introduced in the fields of education, entertainment, medicine, and rehabilitation, such that close contact between robots and humans in daily life is now more widespread [1]. In an environment where humans and robots coexist, static or dynamic collisions with robots can be a source of injury, including shock, clamping, squeezing, and skin injuries. The most common injuries caused by industrial robots are clamping and squeezing injuries.

Therefore, robotic safety has become an important target for research, as robots that share working and living spaces with humans continue to emerge [2–4]. Safety standards for conventional industrial robots are specified in the International Organization for Standardization (ISO10218), which provides guidelines to ensure safe designs and adequate protective measures, as well as operating information pertinent to industrial robots [5]. ISO13482 has recently been published, and addresses safety requirements for service robots. Draft verification and validation standards are currently under review [6]. The University of Mainz, Germany, has determined pressure pain thresholds for different parts of the body in the context of quasi-static collisions between humans and robots, and their findings informed ISO Technical Specification (ISO/TS15066). The rationale for ISO/TS15066 citing a pressure pain threshold rather than an injury threshold is as follows. First, there are no accurate data on mild injury thresholds, and experiments to obtain such data have various practical limitations, including ethical issues. Second, pain thresholds are lower than mild injury thresholds. Therefore, a pain threshold can be used as a safety criterion, assuming that external pressure not exceeding the pressure pain threshold will not cause any physical injury.

In this study, we assessed the pressure pain thresholds of adult males. Linearly increasing pressure was applied to the participants using home-made equipment, with the pressure pain thresholds measured by a fully automated algometer. Pressure pain thresholds were measured in 90 healthy participants at 15 body sites, and pain thresholds at the various sites were examined according to age and body mass index (BMI); whether skin injury had occurred at each pressure pain threshold was also determined.

## Methods

### Pain threshold assessment equipment

**Fig 1:**
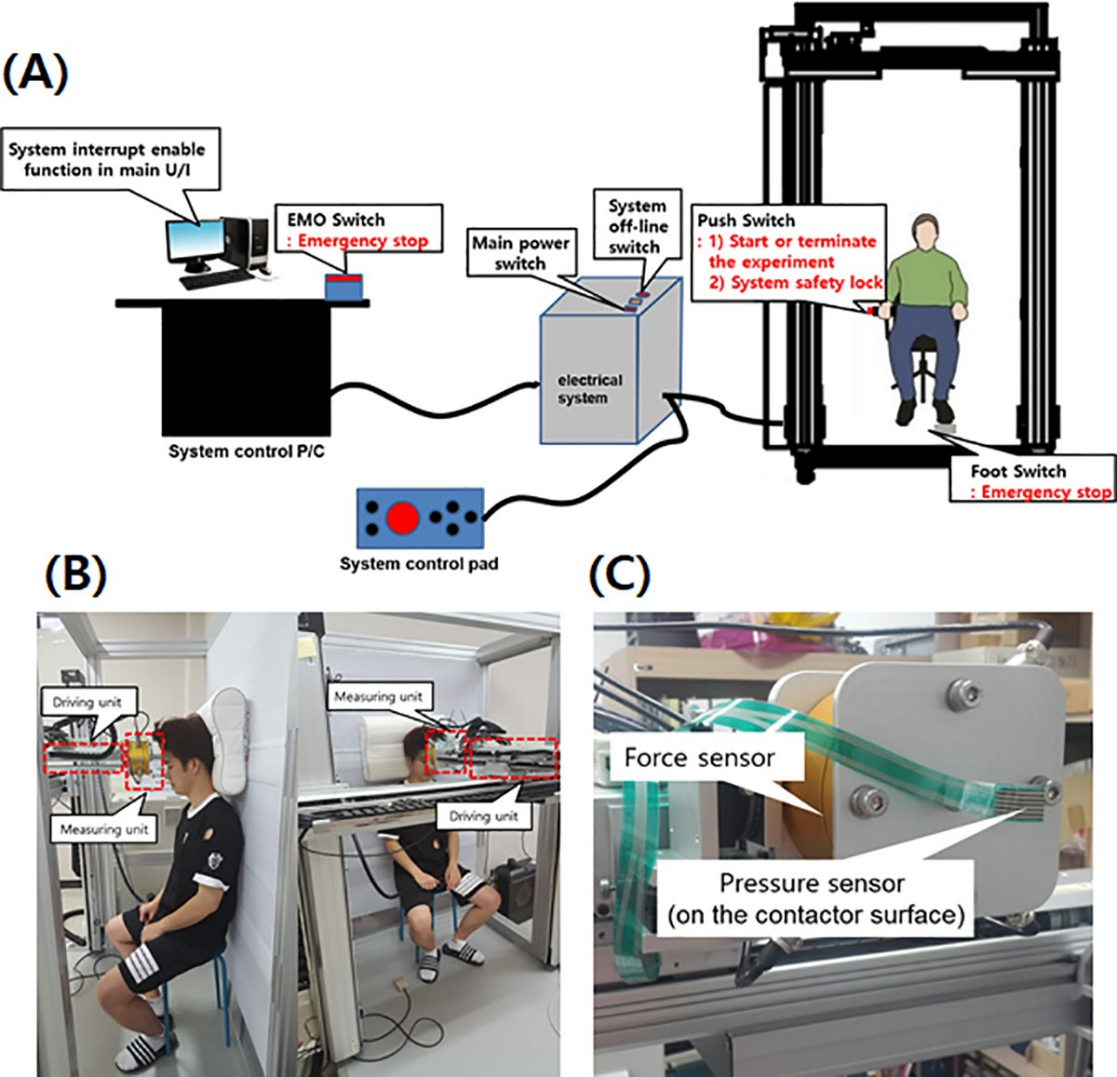
(A) Pressure-generating and measuring equipment, consisting of a driving unit, a measuring unit, and a control program unit. (B) Operating unit. (C) Contactor.

The pressure pain thresholds were measured using a home-built pressure induction device. As shown in Fig 1A, the device consists of a driving unit, a measuring unit, and a control program unit. The driving unit moves the contactor, which applies pressure to the subject (Fig 1B). The measuring unit is composed of force and pressure sensors. A force applied to the subject is measured by a force sensor in three axes. A film-type pressure sensor attached to the end of the contactor measures the magnitude of the pressure applied to the subject. The contactor is a rounded rectangle 1.96 cm^*2*^ in size (Fig 1C).

The measuring sequence is as follows. (1) The driving-unit and contactor are moved to certain points that are described in international standard ISO/TS 15066 by a trained operator. The current position, force, and pressure were set to zero. (2) When the safety function is deactivated by both the subject and the operator, the contactor moves toward the subject. (3) The experiment is terminated when the subject feels critical pain or pressure. At that point, the driving unit moves back to the initial position and the safety function is activated.

**Fig 2:**
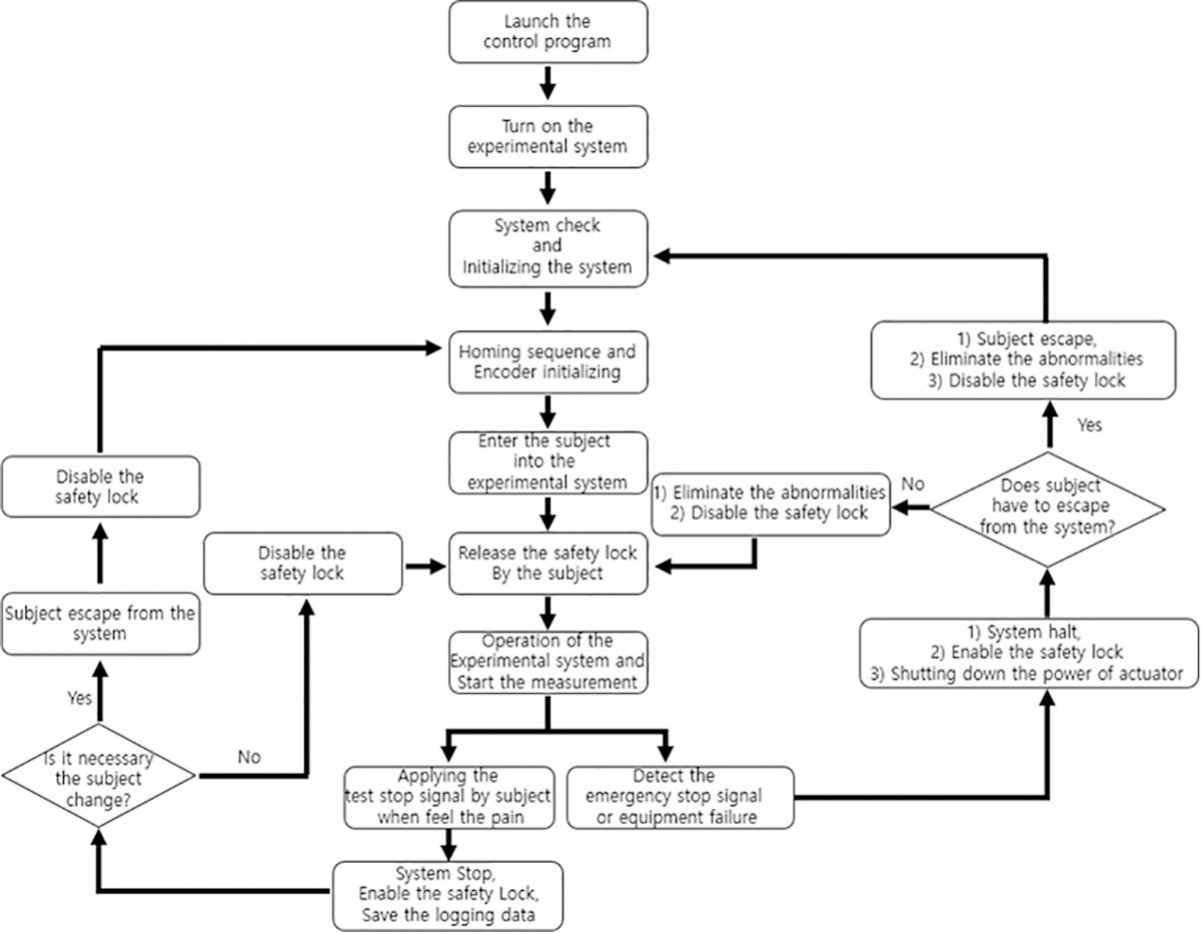
Operating protocol for the pressure pain threshold measurements.

The control system is a program that controls the operation of the contactor and obtains the pressure measurement. The control board consists of a digital signal input terminal for the sensor and servo system monitoring and operation of an emergency safety switch, a digital signal output terminal for servo system control, a D/A converter for motor control, and a high-speed counter to receive encoder signals from the drive shafts. The equipment was operated according to the protocol shown in Fig 2. As there was a possibility of injuring the participants during the experiment, the safety of the equipment was first verified using a test dummy. In addition, the equipment was used only by a trained operator.

### Participants

Ninety healthy subjects participated in this study. All participants were males aged 20–50 years. Participants with eczema, *d*egenerative disk disease, or food or skin allergies were all eligible for inclusion. One participant was allergic to drugs and another to contrast agents; no participant had a metal allergy. We excluded participants with seizure disorders or acute, chronic, or mental illnesses. Participants with cardiac pacemakers, artificial (metal) joints, or metal prostheses were also excluded. Basic physical data were collected for all participants, including height, weight, and BMI, and verbal assessments of health status were conducted. Detailed information on the participants is provided in Table 1. This study was approved by the Institutional Review Board (KHUH 2016-10-022-007). All participants provided written informed consent. The participants were recruited through advertising.

**Table 1:** Patient characteristics.

Characteristics	Description
Number of participants (n)	90
Sex	Male
Age	20 to 50 year (average 28.9 year) 20 to 29 year (n = 60), 30 to 50 year (n = 30)
Weight	48 ~ 110 kg (72.8 ± 11.9 kg)
Height	163 ~ 190 cm (174.8 ± 5.6 cm)
BMI	17.6 ~ 36.4 (23.8 ± 3.5)

### Participant pre-education

Prior to the experiment, the purpose and methodology of the study were explained to the participants, including the pain measurement sites and the order and number of measurements to be taken. A visual analog scale (VAS) was employed to measure the degree of pain. As the experience of pain differs among individuals, the VAS scoring criteria were explained to the participants in detail. A VAS score of 0 denoted no pain and a score of 10 indicated unbearable pain. VAS scores of 1–4 corresponded to mild pain, 5–6 to moderate pain, and 7–10 to severe pain. The pain threshold for static contact corresponded to a VAS pain score of 4–5, i.e., a score of 4–5 was taken as the point at which the sensation of pressure turned into pain. The following examples of the pain threshold were shown to the participants prior to the pain measurements.

- I can tolerate a little more, but I feel pain.
- If pain severity is expressed graphically, the pain threshold is the point at which the slope increases rapidly.
- When receiving a massage, the pain threshold is the point beyond which, if the pressure continues, you feel that you will be injured.
- A VAS pain score of 4–5 corresponds to half of the peak pain level caused by shutting a toe in a door.

### Selection of pain assessment sites

**Fig 3:**
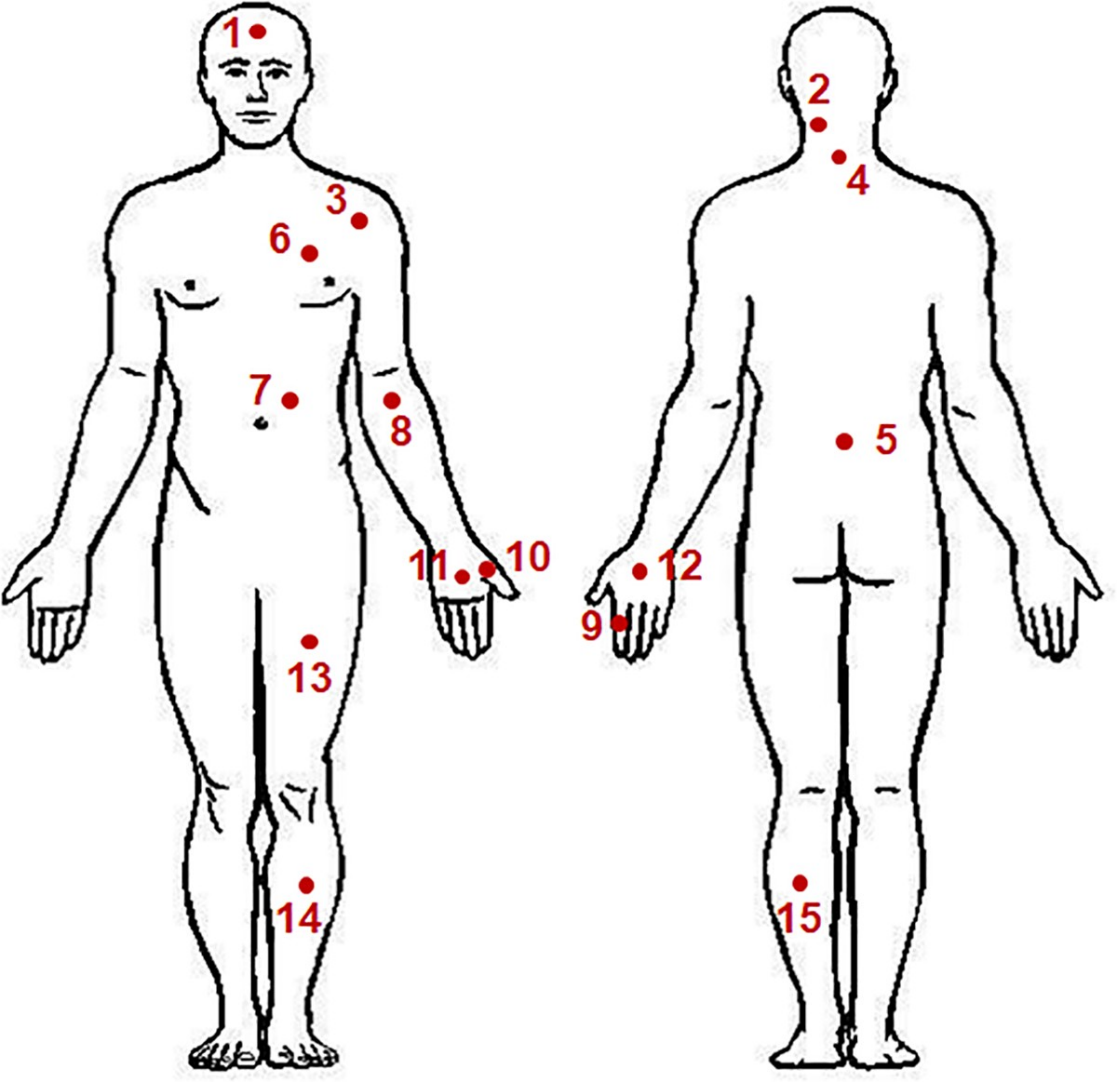
Pressure pain threshold measurement sites on the body.

The pain measurement sites are shown in Fig 3, according to the method employed at the University of Mainz. The 15 measurement sites were as follows: forehead, neck muscle, spinous process C7, shoulder joint, spinous process L5, breast muscle, abdominal muscle, arm nerve, index finger pad, ball of the thumb, palm of the hand, back of the hand, thigh muscle, shin, and calf muscle. Of the 29 measurement sites used by the University of Mainz, all facial areas except the forehead were excluded from this study to avoid damaging thin skin layers. All measurement sites were on the non-dominant body side, i.e., on the left for a right-handed person and on the right for a left-handed person. The location for placing the pressure sensor for each measurement site was selected in consultation with a doctor of rehabilitation medicine. Criteria for each body site and the precise location of the measurements are detailed in Table 2.

**Table 2:** Pain assessment sites in both front and back of the body.

Measuring sites		Description
1	Forehead	2 cm above eyebrow line in middle of left and right eyebrows
2	Neck muscle	5 cm up from Spinous process C7 and 2 cm to the left side
3	Shoulder joint	2 cm outside of the biceps
4	Spinous process C7	The most protruding site of the neck
5	Spinous process L5	Site where bone is touched at the pelvic bone connection line.
6	Breast muscle	5 cm above nipple. Site where the ribs are not touched
7	Abdominal muscle	5 cm above the navel and 2 cm to the left side.
8	Arm nerve	2 cm below the center of elbow wrinkles
9	Index finger pad and joint	Joint of the second finger from the back of hand
10	Ball of the thumb	Muscles between thumb and wrist folds
11	Palm of the hand	Between finger and wrist fold
12	Back of the hand	When you hold your fist, between your third finger and wrist
13	Thigh muscle	15 cm from the upper end of the knee bone
14	Shin	15 cm below the lower edge of the knee bone, 2 cm to the left from the middle of the lower leg bone
15	Calf muscle	Middle point of the calf muscle (gastrocnemius)

### Data collection procedure

The pressure pain thresholds were determined through the following process.

1. First, the experimenter explained the purpose and method of the experiment in detail to the participant. The participant confirmed that they understood the experiment and decided whether or not to participate; the experimenter then guided them to complete the informed consent form.
2. The participant was educated regarding the VAS scoring system.
3. The experimenter placed a sticker on each pressure measurement site. The participant’s posture was adjusted using the support device so that the skin surface of the pressure measurement site was perpendicular to the direction of compression.
4. The pressure device was moved slowly (1 mm/s) toward the pressure measurement site; pressure was then applied to the site.
5. When the intensity of the pressure was judged equal to the pain threshold, the participant stopped the experiment by pressing a button, and the experimenter recorded the pressure at that moment.
6. The participant was then free to relax, and the pressed area was photographed with a dermatologic camera.
7. There was a 10-min rest period between the measurements obtained at each site. Repeat (3) to (6) for the other sites.
8. Measurements were repeated in cases of inaccurate results. Following the experiment, participants were observed for at least 15 min to confirm the absence of any adverse effects at the pressure-measurement site and were then free to leave.

### Statistical analysis

The reliability of the measured results was determined using intra-class correlation coefficients (ICCs). A goodness-of-fit test was performed to determine the distribution of the pressure pain threshold according to body site. Multivariate log-normal regression was performed to assess the relationships between pressure pain threshold, age, and BMI. The statistical analyses were conducted using SPSS (ver. 23.0; IBM Corp., Armonk, NY, USA) and R software (ver. 3.4.3; R Development Core Team, Vienna, Austria). The data are expressed as means ± standard deviation. P-values < 0.05 were statistically significant.

## Results

### Pressure pain thresholds

**Fig 4:**
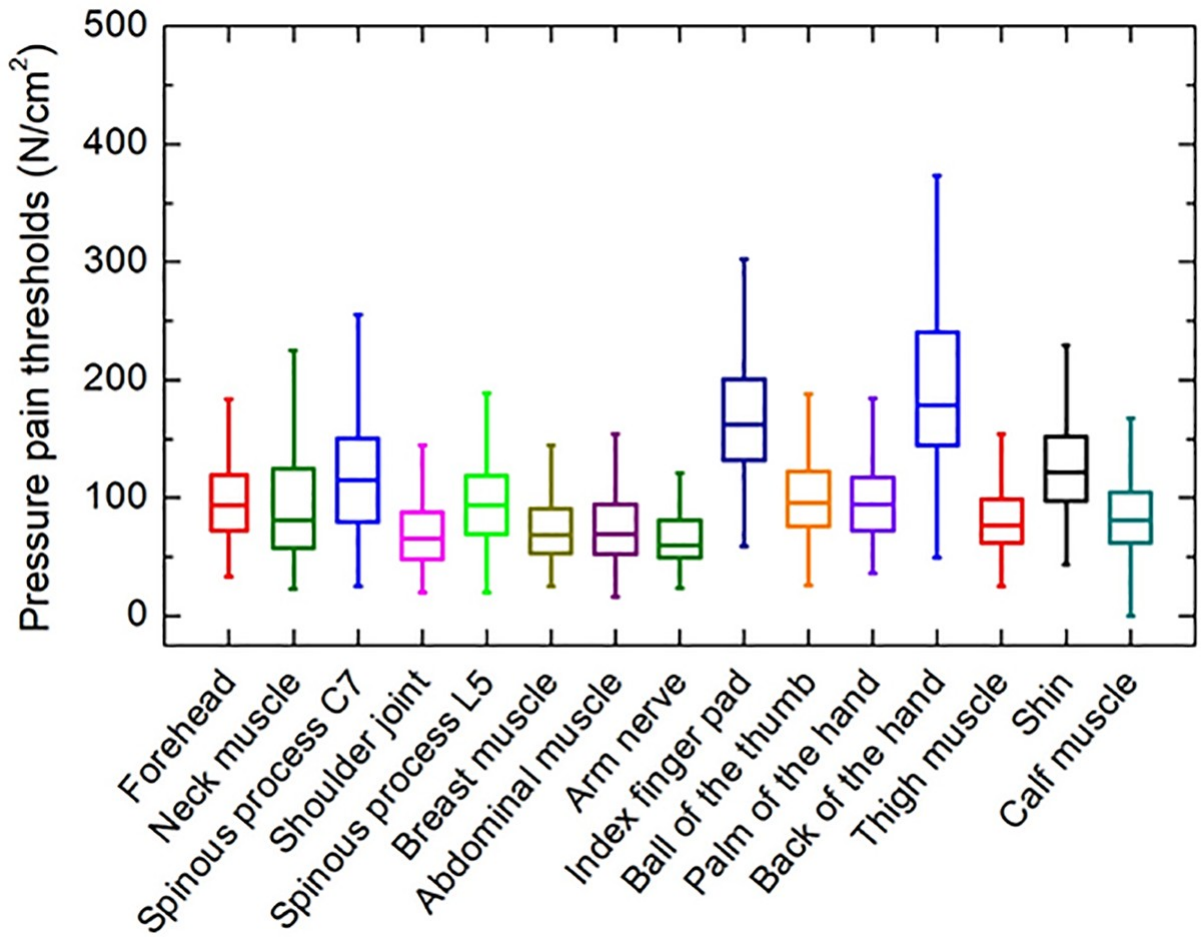
Pressure pain thresholds for 15 body sites; mean values of three repeated measurements at each site for 100 participants.

The pressure pain thresholds of all measurement sites are shown in Fig 4. For each participant, the mean pain thresholds of all sites were calculated based on three repeated measurements. As expected, the pressure pain thresholds varied widely among the measurement sites. Joint, muscle, and nerve sites showed relatively low thresholds of 65.1–87.1 N/cm^2^. Moderate pain thresholds of 100–120 N/cm^2^ were observed at the sites on the forehead, neck muscle, ball of the thumb, and shin. Relatively high thresholds (i.e., > 160 N/cm^2^) were observed at the sites on the finger pad and back of the hand. The lowest threshold was 65.1 ± 22.6 N/cm^2^ at the arm nerve site, and the highest was 196.1 ± 85.8 N/cm^2^ at the back of the hand. The pressure pain thresholds showed a wide distribution at all body sites. Mean threshold values and percentile ranges for all measurement sites are listed in Table 3. The results of three participants for sites 1–7 were excluded because their data were saved incorrectly due to a system error.

**Table 3:** Pressure pain threshold which is arithmetic mean from 3 repeated measurements. Both percentile and quartile for arithmetic mean values.

Measuring sites				Pressure pain threshold [N/cm^2^]								
		n	Mean	Min.	P5	P10	Q1	Q2	Q3	P90	P95	Max.
1	Forehead	87	101.7	47.1	58.9	62.8	72.5	93.9	123.8	150.0	167.3	213.6
2	Neck muscle	87	108.2	24.4	39.1	46.9	62.4	86.4	133.7	203.0	238.5	447.0
3	Spinous process C7	87	124.6	50.2	59.7	67.9	86.2	114.6	153.9	197.2	226.1	294.0
4	Shoulder joint	87	78.4	25.5	35.879	38.4	51.9	65.8	92.9	120.1	137.4	418.6
5	Spinous process L5	87	96.6	32.4	46.5	53.7	75.4	95.7	114.7	133.9	145.5	253.3
6	Breast muscle	87	78.4	34.9	40.5	42.0	54.9	67.5	95.3	110.1	138.6	380.5
7	Abdominal muscle	87	77.4	19.3	34.4	42.1	53.9	71.9	91.0	121.6	137.0	249.9
8	Arm nerve	90	64.9	33.9	39.2	42.2	50.8	60.2	77.5	91.7	98.4	126.0
9	Index finger pad	90	169.1	59.8	104.4	119.0	137.8	160.3	201.2	236.2	241.6	288.8
10	Ball of the thumb	90	104.8	33.8	56.8	60.5	78.9	97.4	119.4	171.7	185.5	228.6
11	Palm of the hand	90	97.8	43.8	55.5	59.2	73.9	91.6	118.6	146.3	157.5	198.1
12	Back of the hand	90	196.2	73.7	98.6	129.4	151.1	188.5	233.9	287.9	323.2	428.9
13	Thigh muscle	90	81.3	30.6	48.3	53.6	60.7	75.9	93.7	121.2	130.8	182.2
14	Shin	90	128.2	45.6	68.2	72.8	96.5	123.1	144.8	188.4	212.7	309.4
15	Calf muscle	90	84.4	33.1	44.7	50.9	62.8	79.8	98.9	126.7	138.2	226.5

### Pressure pain thresholds by age and BMI

**Fig 5:**
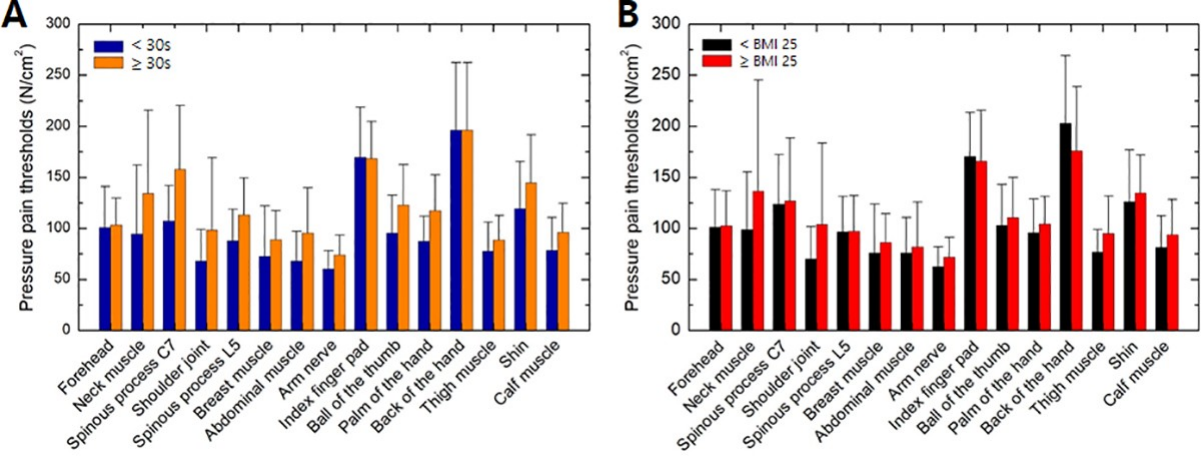
Variations in pressure pain thresholds by (A) age and (B) body mass index (BMI).

The pressure pain thresholds were evaluated according to age and BMI. Fig 5(A) shows the correlation between age and pressure pain threshold for all measurement sites. The participants were divided into two groups according to age. There were 60 participants aged < 30 years (23.8 ± 2.6 years) and 30 aged > 30 years (38.5 ± 8.0 years). The pressure pain thresholds of the participants < 30 years of age were lower than those of participants aged > 30 years at all measurement sites. Spinous process C7 showed the largest difference in threshold by age group (32.1%), followed by the shoulder joint (31%), and neck muscle (29.4%). The forehead, index finger pad, and back of the hand showed only slight differences between age groups (< 3%). The differences in threshold between age groups were in the range of 12–28% at all other sites.

Fig 5(B) shows the correlation between BMI and pressure pain threshold for all body sites. The BMI of each participant was calculated according to their height and weight, which were measured before the experiment. The standard BMI categories for adults were used, as follows: underweight, BMI < 18.5; normal or healthy weight, BMI 18.5–24.9; overweight, BMI 25.0–29.9; and obese, BMI > 30.0 kg/m^*2*^ [7]. Among all participants, three were underweight, 65 were of normal or healthy weight, 15 were overweight, and seven were obese. We divided the participants into two groups according to a BMI cut-off of 25 kg/m^*2*^, which is considered to be the borderline between normal and overweight. The pressure pain thresholds of the high-BMI (> 25 kg/m^*2*^) group were higher or similar to those of the low-BMI (≤ 25 kg/m^*2*^) group, except at the back of the hand and the index finger. The largest differences in pain threshold by BMI group were observed at the shoulder joint and neck muscle, where the thresholds of the high-BMI group were 32% and 27% larger than those of the low-BMI group, respectively. The high-BMI group had a 15% lower pain threshold at the back of the hand. No significant differences were observed in the pressure pain threshold between the BMI groups at the other body sites.

### Mild skin damage

**Fig 6:**
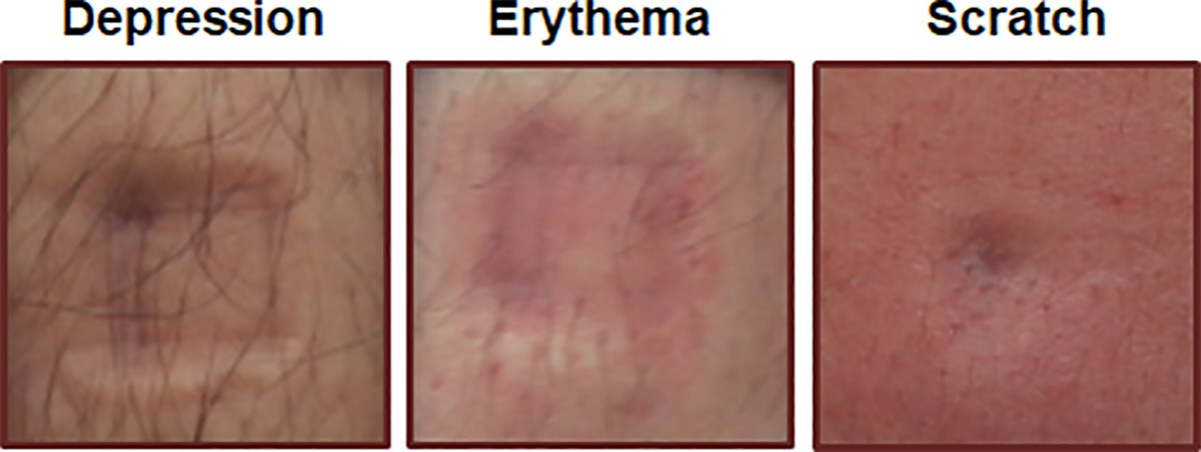
Skin changes after pressure pain threshold measurements. Mild changes, including depressions, erythema, and scratch, were primarily observed.

Skin changes induced by pressure were analyzed by a dermatologist. A total of 4,050 photographs were taken of the measurement sites immediately after the experiment (90 participants × 15 measurement sites × 3 repeated measurements). No severe skin damage was observed in any participant; damage tended to be mild, such as depressions, erythema, and/or edema. Fig 6 shows a representative photograph of skin with mild damage. The most common type of damage was depressions (43.8%), followed by erythema (30.8%). Only four cases of scratching were observed (in three participants). In the majority of cases, skin damage disappeared after a few minutes to several hours. There were no reports of damage persisting until the following day.

## Discussion and conclusion

The main purpose of this study was to inform safety standards for robots that interact closely with humans by quantifying pressure pain thresholds in collision incidents. Pain thresholds were determined at 15 body sites in 90 males. The thresholds were in the range of 65.1–196.1 N/cm^2^ and varied among individuals and by measurement site, age, and BMI. Pain is a very subjective experience that can vary according to internal and external factors, such as the subject's physical condition and emotional state, the order in which measurements are taken, and the time of measurement. Therefore, the reliability of the measured results was verified by analytic and statistical methods in this study.

**Fig 7:**
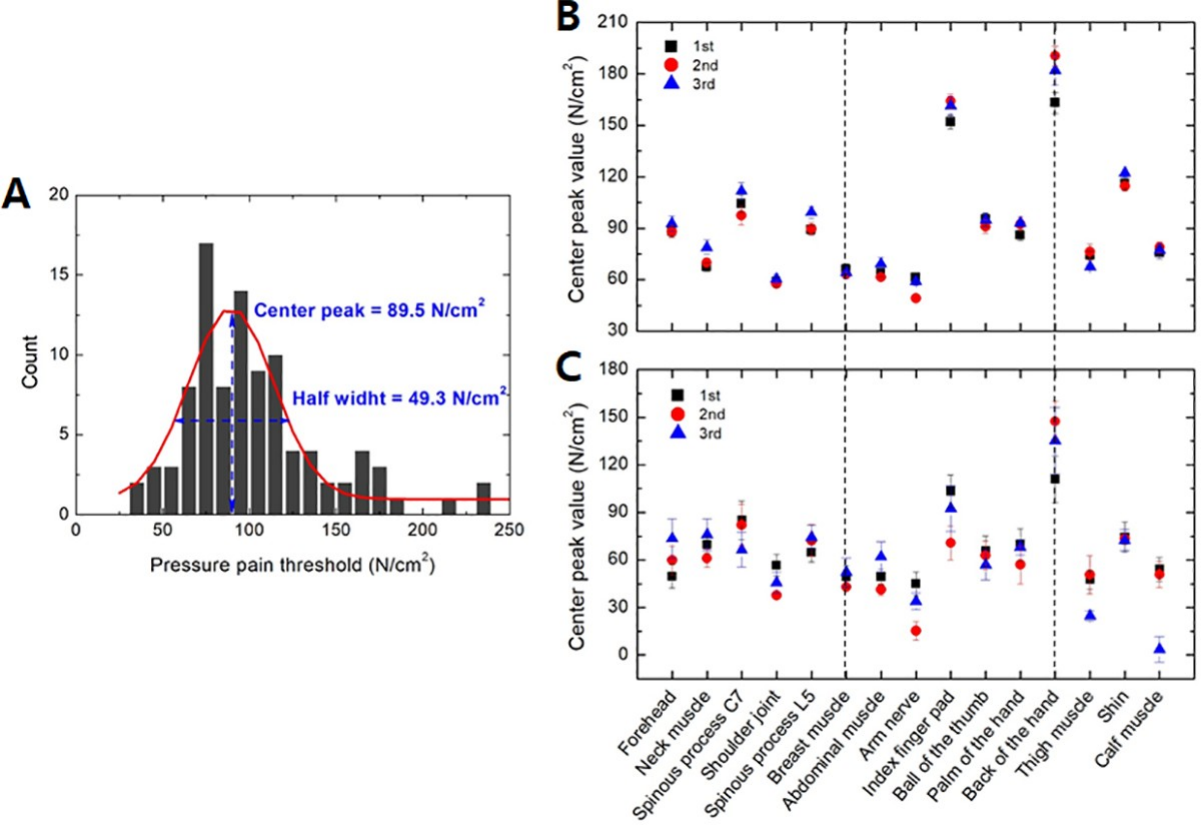
(A) Distribution of pressure pain threshold measured at the forehead. (B) Peak values and (C) half-width determined from the Gaussian fit of the thresholds of 100 participants.

First, repeatability within the test was evaluated by comparing the distribution of the pressure pain thresholds obtained for three repeated measurements. Fig 7(A) shows the pain threshold values obtained at the forehead during the first measurement. As the thresholds showed a normal distribution, they were analyzed by Gaussian modeling. The red line indicates the Gaussian fit to the data. The peak values of the Gaussian fit for the three measurements obtained at each site are shown in Fig 7(B). These values were similar among nine of the measurement sites, including the forehead. A difference in pain threshold was detected among the three measurements obtained at the index finger pad and the back of the hand. At these sites, the first measurements showed the lowest values, with the pressure pain threshold being slightly increased on the second and third measurements. However, the differences were not significant. Fig 7(C) shows the half-width of the Gaussian function, which indicates individual differences. Notably, the half-width values showed the same dependency on body site as the peak value. The half-width value was lowest at the shoulder joint (which also had the smallest peak value), and largest at the back of the hand (which also had the largest peak value). There was a difference in the half-width value of the three repeated measurements on the forehead, arm nerve, index finger pad, back of the hand, thigh, and calf muscle, but there was no such difference at any other measurement site. This finding indicates that the wide distribution of pressure pain thresholds shown in Fig 4 was influenced more by individual differences among participants than by the number of measurements.

**Fig 8:**
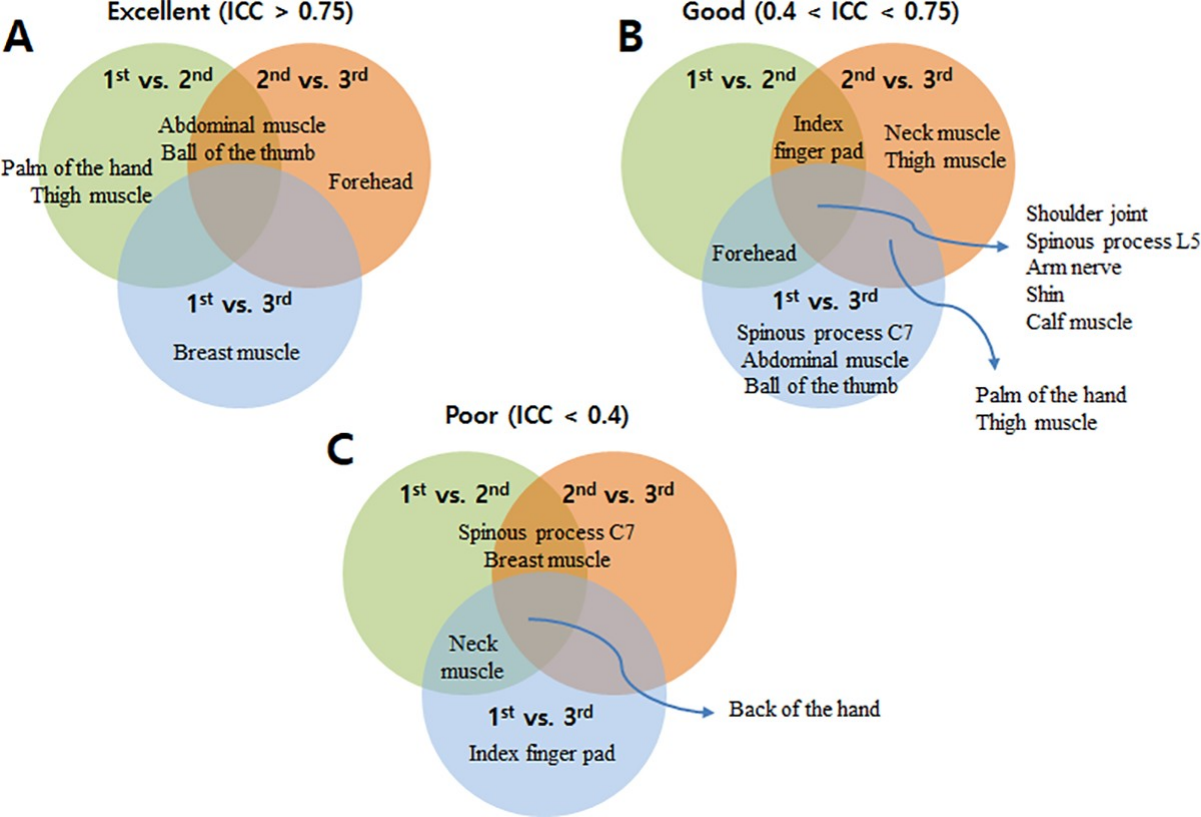
Intra-class correlation coefficients (ICCs) between the first, second, and third measurements at each site. The correlations were classified as good (ICC ≥ 0.75), fair-to-good (0.40 ≤ ICC < 0.75), or poor (ICC < 0.40).

Test repeatability was also assessed by the ICC, which is a widely used statistical method introduced by Shrout and Fleiss in 1979 [8]. Among the several types of ICC statistics proposed by Shrout and Fleiss, ICC agreement was deemed appropriate for the purposes of this study and thus was applied to index the repeatability of the measurements [9]. According to the generally accepted standards, an ICC ≥ 0.75 denotes an excellent correlation, an ICC 0.40–0.75 corresponds to a fair-to-good correlation, and a correlation of ≤ 0.40 is considered poor. The ICCs in this study were calculated along with their 95% confidence intervals. The ICCs varied both by measurement site and comparison class. As shown in Fig 8A, the abdominal muscle, ball of the thumb, forehead, palm of the hand, thigh muscle, and breast muscle sites showed excellent repeatability depending on the comparison class, with ICCs > 0.75. The majority of the measurement sites (13 of 15) showed good repeatability depending on the comparison class (Fig 8B). In particular, five body sites (shoulder joint, spinous process L5, arm nerve, shin, and calf muscle) showed good repeatability in all intra-class correlations. The back of the hand showed poor repeatability in all intra-class correlations.

Second, a goodness-of-fit test was performed to determine the distribution of the pressure pain threshold according to body site. The goodness-of-fit tests were conducted according to five theoretical probability distribution functions: the Kolmogorov–Smirnov statistic, the Cramer–von Mises statistic, the Anderson–Darling statistic, Akaike’s information criterion, and the Bayesian information criterion [10–12]. The extent to which the pressure pain thresholds of the various measurement sites showed a normal distribution, log-normal distribution, gamma distribution, uniform distribution, or Weibull distribution was examined. The thresholds of all sites, except spinous process L5, were log-normally distributed. Therefore, the analysis described below was conducted according to a log-normal distribution.

**Fig 9:**
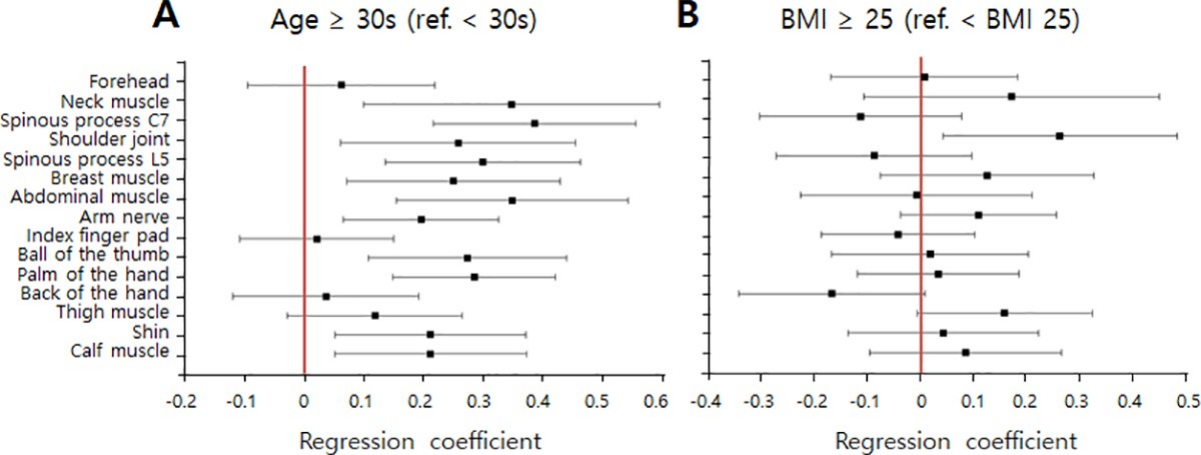
Parameter estimates and 95% confidence intervals for multivariate log-normal regressions examining pressure pain thresholds as a function of age (A) and BMI (B).

Third, a multivariate log-normal regression was performed to identify and quantify the associations between the pressure pain threshold, age, and BMI. Fig 9A shows the parameter estimates and 95% confidence intervals for the multivariate log-normal regression to evaluate the pressure pain threshold as a function of age. At all 15 measurement sites, the pain threshold was positively associated with age; the association failed to reach significance at the forehead, index finger pad, or the back of the hand. Fig 9B shows the results of the regression analysis between the pressure pain threshold and BMI; both positive and negative associations with BMI were observed and varied according to the measurement site. None of the associations were significant.

In conclusion, this study was designed to inform safety standards for collaborative robots. Pain thresholds relevant to potential human-robot collisions were determined and were shown to be in the range of 65.1 ± 22.6 to 196.1 ± 85.8 N/cm^2^. The thresholds depended on individual characteristics, body site, and age.

## References

[1] Ferrández JM, de Lope J, de la Paz F. Social and collaborative robotics. Robotics and Autonomous Systems. 2013;61:659–660.

[2] Jeremie G, Mathilde M, Helene W. Safety-critical advanced robots: A survey. Robotics and Autonomous Systems. 2017;94:43–52.

[3] Gribovskiy J. Halloy JL, Deneubourg FM. Designing a socially integrated mobile robot for ethological research. Robotics and Autonomous Systems. 2018;103:42–55.

[4] Bernhard D, Benjamin B, Sebastian T, Severin K, Stefan R, Peter S. Security for the robot operating system. Robotics and Autonomous Systems. 2017;98:192–203

[5] Roger F. International Organization for Standardization (ISO). Qual Assur J.2004;8:198–206.

[6] Theo J, Gurvinder SV. ISO 13482—The new safety standard for personal care robots. Conference ISR ROBOTIK 2014.

[7] Nuttall FQ. Body Mass Index. Nutrition Today. Nutr Today. 2015;50(3):117–128. 10.1097/NT.0000000000000092 27340299 PMC4890841

[8] Terry KK, Mae YL. A Guideline of selecting and reporting intraclass correlation coefficients for reliability research. Journal of Chiropractic Medicine. 2016;15:155–163. 10.1016/j.jcm.2016.02.012 27330520 PMC4913118

[9] Min K, Yu S, Lee J, Song J, Shim J, Lee H, et al Reliability of fractional anisotropy measurement for children with cerebral Palsy. Neuropediatrics. 2014;45:84–92. 10.1055/s-0033-1357480 24122275

[10] Neetu S, Kanchan J, Suresh KS. Goodness of fit tests and power comparisons for weighted gamma distribution. REVSTAT. 2016;14:29–48.

[11] Jin Z. Powerful goodness-of-fit tests based on the likelihood ratio. J. R. Statist. Soc. B. 2002;64:281–294.

[12] Conover WJ, Kolmogorov A. Goodness-of-fit test for discontinuous distributions. Journal of the American Statistical Association September. 1972;67:591–596.

